# Self-Generated Chemoattractant Gradients: Attractant Depletion Extends the Range and Robustness of Chemotaxis

**DOI:** 10.1371/journal.pbio.1002404

**Published:** 2016-03-16

**Authors:** Luke Tweedy, David A. Knecht, Gillian M. Mackay, Robert H. Insall

**Affiliations:** 1 Cancer Research UK Beatson Institute, Glasgow, United Kingdom; 2 Department of Molecular and Cell Biology, University of Connecticut, Storrs, Connecticut, United States of America; Institute for Systems Biology, UNITED STATES

## Abstract

Chemotaxis is fundamentally important, but the sources of gradients in vivo are rarely well understood. Here, we analyse self-generated chemotaxis, in which cells respond to gradients they have made themselves by breaking down globally available attractants, using both computational simulations and experiments. We show that chemoattractant degradation creates steep local gradients. This leads to surprising results, in particular the existence of a leading population of cells that moves highly directionally, while cells behind this group are undirected. This leading cell population is denser than those following, especially at high attractant concentrations. The local gradient moves with the leading cells as they interact with their surroundings, giving directed movement that is unusually robust and can operate over long distances. Even when gradients are applied from external sources, attractant breakdown greatly changes cells' responses and increases robustness. We also consider alternative mechanisms for directional decision-making and show that they do not predict the features of population migration we observe experimentally. Our findings provide useful diagnostics to allow identification of self-generated gradients and suggest that self-generated chemotaxis is unexpectedly universal in biology and medicine.

## Abstract 2

A combination of experiment and simulation reveals self-generated chemotaxis—an emergent, fundamental, and widespread mechanism of directed cell migration that depends on removal or destruction of the chemotactic signal by the migrating cells themselves.

## Author Summary

Cells move in response to chemical gradients. This is chemotaxis, a fundamental process that is crucial for embryonic development, immune response, and the progression of cancer. How the environment dictates cell movement has been studied extensively, but most studies do not account for how cells alter the environment and the resultant feedback between these two processes. In this study, we describe self-generated gradient chemotaxis, a new paradigm in cell movement that depends entirely on the two-way interaction between cells and their environment. We show that self-generated gradients guide cells over much longer distances than are possible by other means. As these distances are biologically relevant, we conclude that the self-generated gradient is in fact the fundamental chemotactic mechanism in real biological contexts such as immunity and cancer. We show how self-generated gradients work and describe indicators that can be used to identify them.

## Introduction

Eukaryotic chemotaxis is a key mechanism in many biological processes, including wound healing, development, and the metastasis of cancers [1]. In spite of its profound biological and medical importance, we frequently do not know enough about the environments of cells to predict their chemotactic responses. The sources of chemoattractants are often unknown or vague, and we do not always know how cells alter those gradients they do encounter. Attractant sinks are obviously crucial to gradient generation but are rarely analysed [2]. This means we often must make potentially inaccurate assumptions in order to interpret the outcomes of experiments.

Several chemotactic cell types are known to degrade their chemoattractants. For example, we have recently shown that melanoma cells are highly chemotactic to lysophosphatidic acid (LPA) but also efficiently break it down [3]; *Dictyostelium* cells, a widely used model for chemotactic systems, are unable to develop without enzymes that break down cAMP, the principal chemoattractant in this process [4]. There are multiple ways to remove chemoattractants. In growth factor chemotaxis, the ligands are endocytosed and broken down during signalling [5], which can alter extracellular signal levels in a way that stabilises gradients [6]. Other systems use dummy receptors, which have been elegantly shown to be crucial in the case of the zebrafish lateral line primordium [7,8]. It is clear that ligand removal must profoundly affect the behaviour of the systems they are part of, yet these environmental interactions are rarely addressed when investigating chemotaxis.

Here, we focus principally on the influence of chemoattractant degradation. We explore the effect it has on the profile of environmental chemoattractant and, by extension, on the behaviour of cells in two frequently used assays. We go on to describe key behavioural features of this system that can be used to identify its action in other contexts.

## Results

### Self-Generated Gradients Can Drive Cell Motility in Uniform Chemoattractant

In our earlier work, we studied melanoma cells moving up a self-generated gradient [3]. However, melanoma cells move relatively slowly, and LPA is potentially confusing, as it can act as a mitogen as well as an attractant [9], and cells may produce it as well as break it down [10]. For a more global understanding of self-generated gradients, we sought a more straightforward assay. Under-agarose migration of *Dictyostelium* cells is ideal [11]—the agarose restricts convection without greatly affecting diffusion, and the *Dictyostelium* cells move rapidly and are highly chemotactic. Although cAMP is the most widely used attractant, responses to cAMP are developmentally regulated, which means they vary over time. In addition, cells synthesize and secrete cAMP, potentially confusing the interpretation of responses to imposed diffusional gradients. We therefore used folate, the principal attractant for growing cells. Folate—like cAMP in *Dictyostelium*, LPA in cancer cells, and fMLP in neutrophils—is detected by serpentine receptors and G-proteins [12]. Cells can also break down folate using secreted and cell-surface folate deaminase [13], allowing cells to generate folate gradients while responding to them.

**Fig 1:**
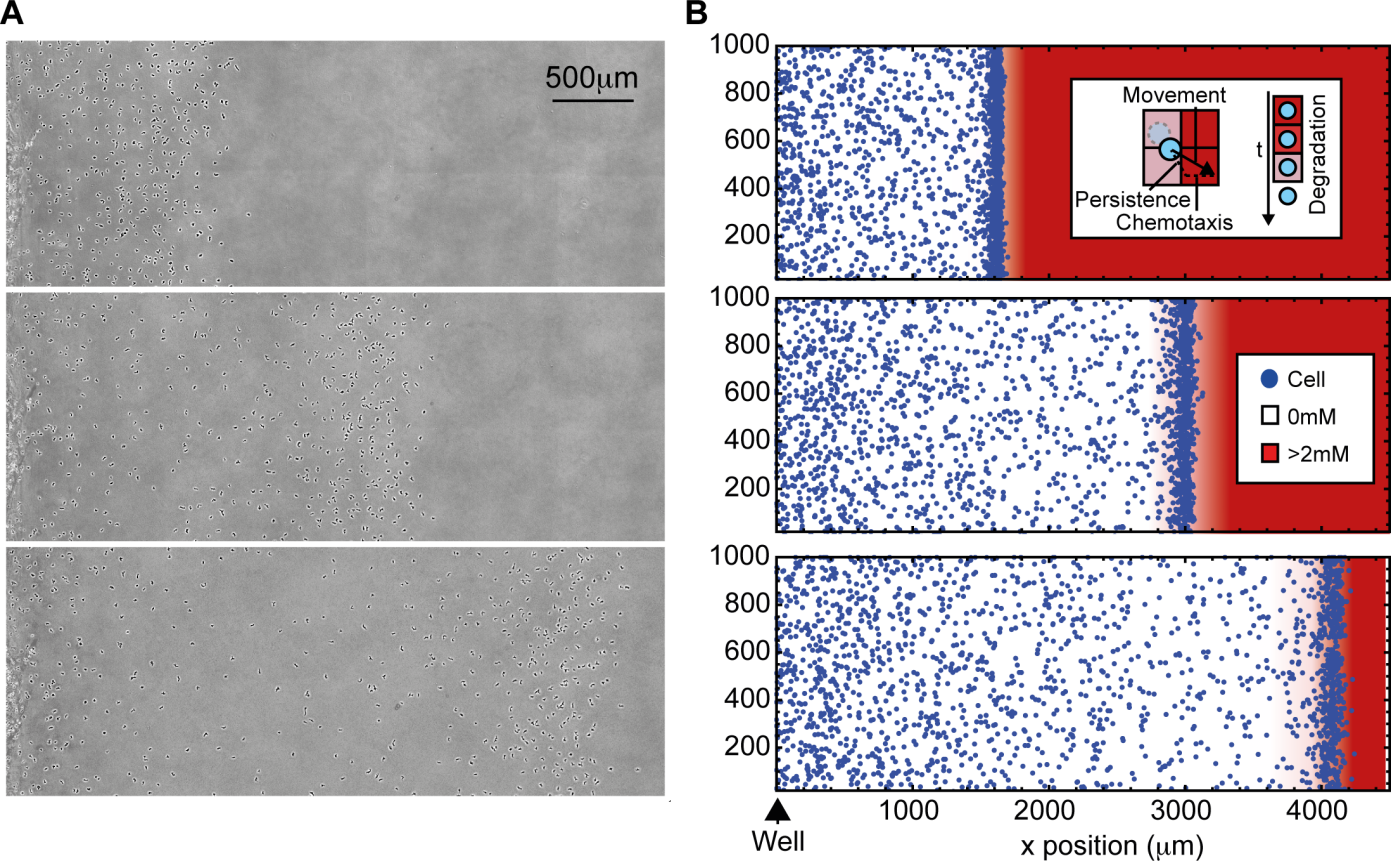
A self-generated gradient model can explain directed motility in a spreading assay. (A) *Dictyostelium* cells migrating in the under-agarose assay. Agarose initially contains 20 μM folate. (B) A simulation of cells migrating in response to a self-generated gradient. (Inset) Cells move in a 2-D persistent random walk, biased by local attractant gradient and degrade local attractant over time. The data for this figure can be accessed at doi: 10.5525/gla.researchdata.252.

Assays of this type have been used elsewhere, particularly in genetic selections [14], but ours was optimised to be robust enough to reveal long-term evolution of cell behaviour. It also—serendipitously—allowed self-generated gradients to be measured directly (see below). Cells were inoculated into a small well cut into an agarose sheet containing 20 μM folate. Initially there is no gradient, but as the cells are tightly localised, they break down the attractant in the surrounding area, forming a gradient that the cells can themselves respond to (Fig 1A and S1 Movie). As expected, we saw motile cells travelling in a directed fashion in 20 μM folate (〈cos **θ**〉 = 0.49 between 1–2 h) and saw no substantial motility without chemoattractant (〈cos **θ**〉 = 0.035 between 1–2 h). However, the chemotaxis was maintained for a longer time and greater distances (7 h and 5 mm, respectively) than most standard chemotaxis assays, such as Zigmond chamber assays [15].

Proving the detailed role of a self-generated gradient is extremely difficult. We typically include a fluorescent molecule of the same size as the attractant as a gradient tracer. However, the tracers are not modified by attractant-degrading enzymes and are thus inappropriate for gradients generated by breakdown. Fluorescently-labelled attractants are no better—while they can be broken down by the physiologically appropriate enzymes, their degradation products remain equally fluorescent, so gradients of attractant bioactivity are not paralleled by gradients in fluorescence. We therefore began our investigation into the detailed behaviour of attractants by creating an agent-based computational model of the process, in which an initially uniform and freely diffusing attractant is degraded by a population of chemotactic cells, which themselves move with a bias up any local attractant gradient (Fig 1B and S2 Movie). Attractant degradation in these simulations is local to each cell, with attractant removed from the immediate vicinity, and follows Michaelis-Menten kinetics, with each cell able to destroy attractant at the same maximum rate. Simulations replicate the attractant-driven motility of the experimental condition, demonstrating that such behaviour can result from a self-generated gradient. The simulations also indicate the probable evolution of the attractant profile, though the details of all such inferences require verification through testing of the model’s predictions on real cells.

### Receptor Occupancy Difference Replicates Behaviour over a Large Concentration Range

**Fig 2:**
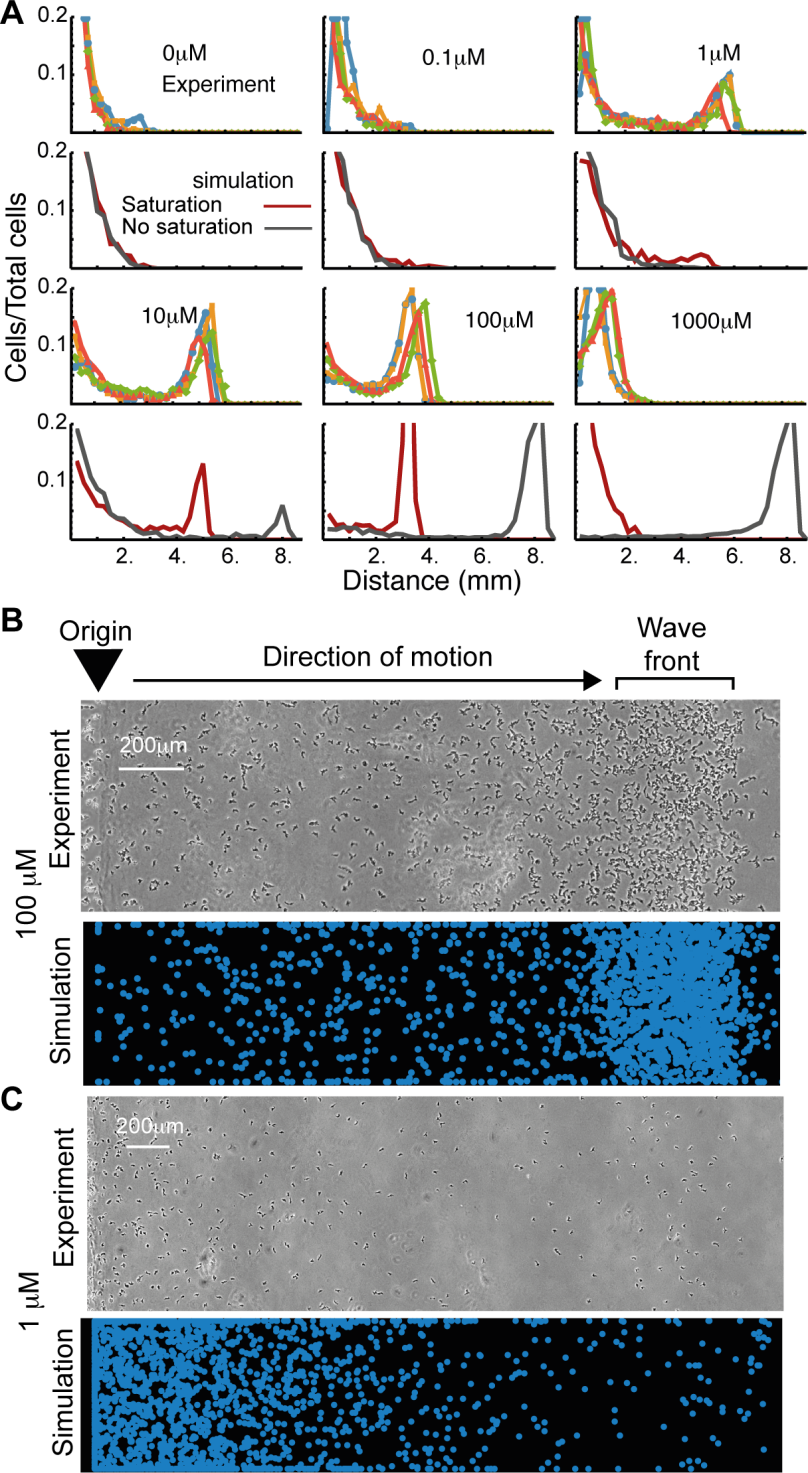
Receptor occupancy coupled with a self-generated gradient reproduces fine details of experimental distributions over a large concentration range. (A) Distribution of the fraction of the population found at different distances from the well for cells in four under-agarose experiments performed on two separate days, and simulations (red and grey lines) over a range of folate concentrations. Simulations in grey do not account for saturating receptor kinetics. Simulations in red do. (B) Images taken of a 100 μM folate under-agarose assay after 6 h (top) and a simulation of the condition (bottom). (C) As with B, but for the 1 μM folate assay. doi: 10.5525/gla.researchdata.252.

We simulated self-generated chemotaxis across a wide range of chemoattractant concentrations (Fig 2). One feature of these simulations is striking: at higher concentrations, in which there was substantial motility, we observed a population density peak at the leading front of the migration out of the well. We then performed the under-agarose assay over the same range of attractant concentrations. These assays confirmed the model’s prediction of a density peak (Fig 2B). The peak was more pronounced at higher concentrations of attractant in both simulations and experiments.

Results from a simple model—in which chemotaxis simply responds to the local attractant gradient—replicated the existence of a density peak at the front of the migratory wave but predicted the distance travelled by the wave poorly, particularly at higher concentrations (Fig 2A, grey lines). We therefore improved the guidance model by using estimated receptor occupancy difference across the cell (as described by Zigmond in [15]) rather than absolute attractant difference to guide movement. Given a physiologically realistic dissociation constant for *Dictyostelium* folate receptors (K_*d*_ ~20 nM, for the B receptor in [16]), this refined model efficiently reproduces the behaviour of the spreading assays across the whole range of explored concentrations (Fig 2A, red lines), with the simulated population front reaching the same distance as its experimental counterparts after a set time in each condition, and again featuring a densely populated travelling wave at high concentrations. The low chemotaxis at extremely high concentrations is caused by receptor saturation in these simulations—cells cannot break down folate quickly enough, so receptors at the front and rear are fully bound, and no gradient can be resolved. This observation cannot be explained by chemokinesis—we would expect migration to still be induced if migration was simply driven by receptor occupancy. A chemokinetic model would also not explain the formation of the population density peak, as induced (but randomly directed) migration would give rise to a Gaussian profile.

### Only Leading Front Cells Perceive Chemoattractant Gradients

The front of cells leading the migration out of the well could form the density wave for several different reasons: different initial conditions, evolving differences in environmental conditions, or (in real cells) phenotypic diversity. We therefore interrogated the simulations to investigate behavioural differences near to and far from the well.

**Fig 3:**
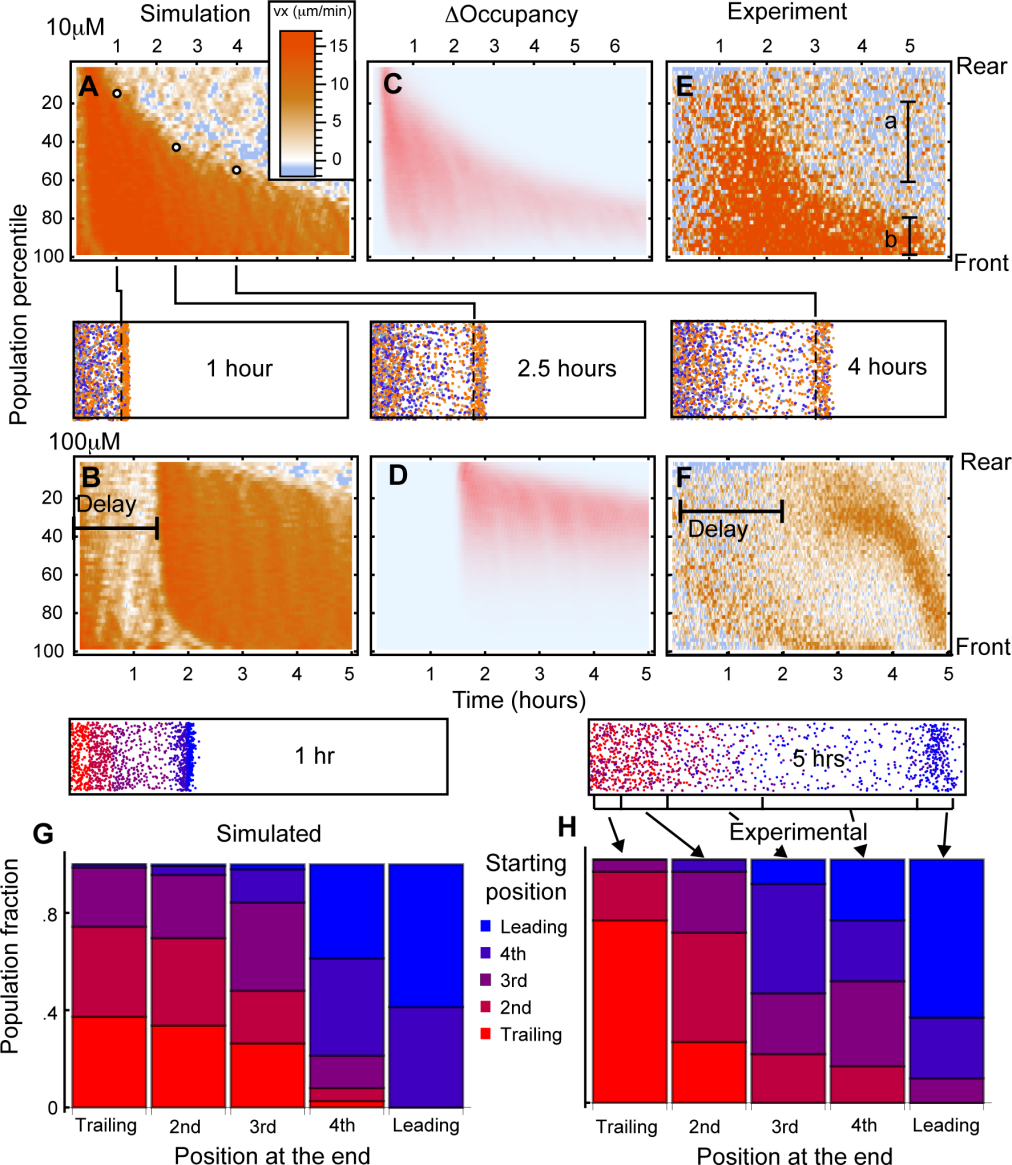
Leading edge dynamics are caused by singular exposure to chemoattractant. (A, B) The x component of cell velocity across the population for 10 μM (A) and 100 μM (B) simulations. The population is sorted by proximity to the well and binned into 2% sections. The population percentile is shown on the *y*-axis, with cells nearest the well shown at the top and cells furthest from the well shown at the bottom. Time progresses along the *x*-axis. Colours indicate velocity directed away from the well, as is indicated in the legend. White dots in (A) are placed at the divide between chemotaxing and nonchemotaxing cells for three different time points and correspond to the positions of the dotted lines in the illustrative stills of a 10 μM simulation at these time points. S3 Movie demonstrates the construction of (A). (C, D) Receptor occupancy differences for A and B. (E, F) Directed velocity of cells in 10 μM (E) and 100 μM (F) folate experiments. The movement of cells in the region labelled ‘b’ showed clear directionality, where those in the region labelled ‘a’ were indistinguishable from 0. (G, H) Comparison of starting positions of cells with their positions after 5 h for simulated (G) and real (H) cells. Bars are arranged according to the position of cells at the end of the experiment, with cells finishing in the trailing quintile represented in the leftmost bar and those further forward shown to the right, up to the leading quintile in the rightmost bar. Colours indicate the starting quintile of the cells, as shown in the left-hand still of the simulation, with red indicating those cells that began at the rear through to blue indicating those that began at the front. doi: 10.5525/gla.researchdata.252.

**Fig 4:**
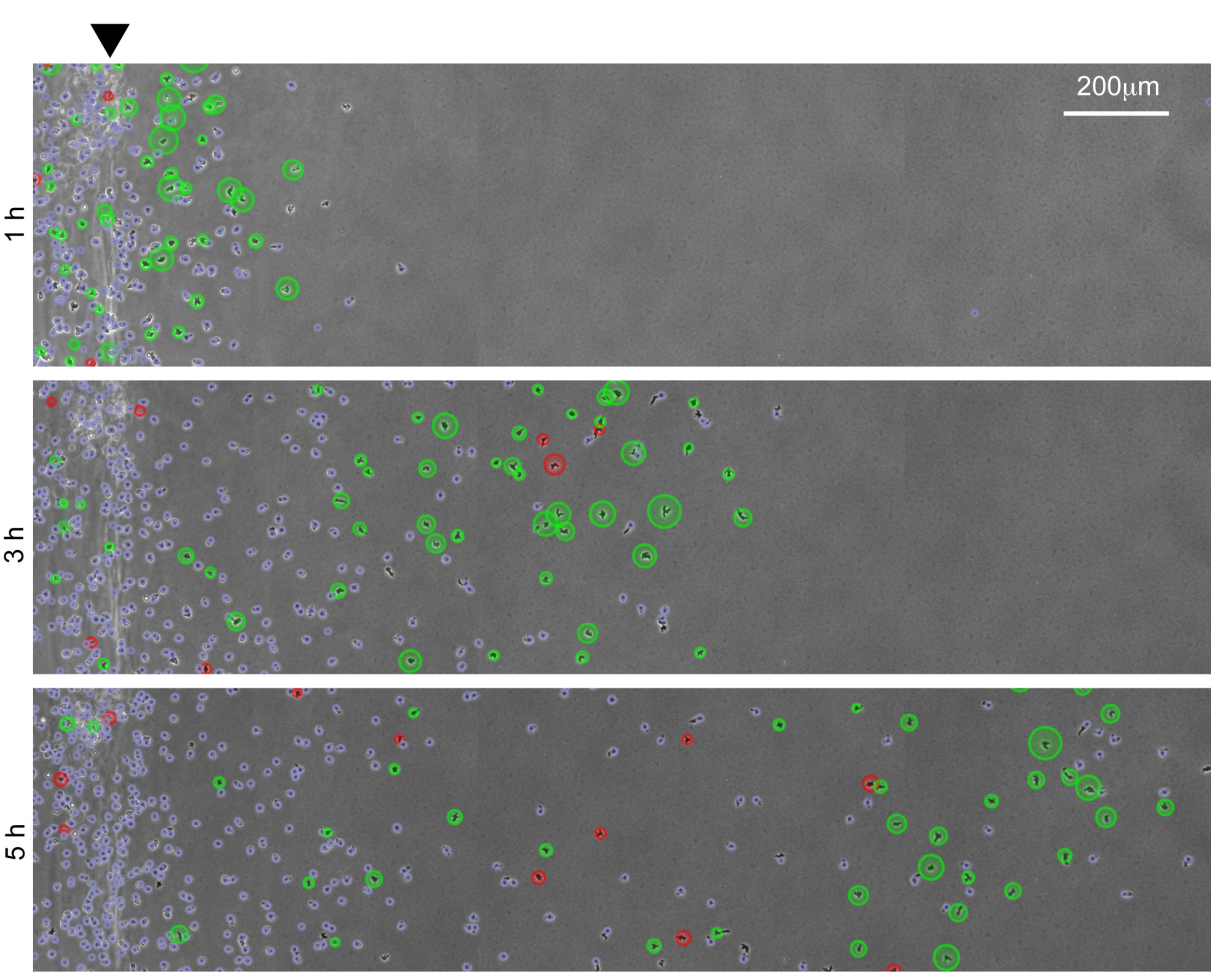
Two distinct behaviours are spatially separated in the migrating population. Cells migrating in the 10 μM folate under-agarose assay. Those circled in green are travelling away from the well (x component of velocity >1 μm/min), those in red are travelling toward it. Larger circles indicate faster movement in the appropriate direction. Cells marked in blue show no substantial movement either way. doi: 10.5525/gla.researchdata.252.

We plotted the x-component of the velocity vector (the component directed away from the well) of every cell over time (Fig 3A–3F). Fig 3A, 3B, 3E and 3F are counterintuitive and thus rather difficult to interpret; we have clarified them by showing the evolution of such a graph in S3 Movie. In simulations in which there was motility, there was a clear, and surprisingly discrete, separation between directed, chemotaxing cells in the density wave at the front (orange) of the population and undirected, randomly walking cells (blue/orange noise), accounting for the remainder (Fig 3A and 3B). In the simulation, all cells are identical; the ones in the population density wave differ from those the wave leaves behind only in their receptor occupancy difference (Fig 3C and 3D). This implies that the density wave is defined by cells’ access to a chemoattractant gradient. We measured the x component of velocity of >500 real cells in the under-agarose assay and compared it with the simulation. The behaviour was remarkably similar (Fig 3E and 3F), with the same spatial divide as seen in the simulation (see also Fig 4 and S4 Movie). We confirmed that the rearmost cells were genuinely nondirected using a location test on the x component of the velocity. In a 15-min window centred at 5 h into the 10 μM assay, the rear population did not move significantly away from the well (Fig 3E, label a—cell population between the 2nd and 6th deciles, mean μ = 1.04, one sample *t* test, *p* = 0.152), whereas the cells at the front were clearly directed away from the well (Fig 3E, label b—population between the 8th and 10th deciles mean μ = 13.6, *p*~10^−19^). Thus, self-generated gradients split the populations of cells that respond—the front fraction chemotaxes, while the rear fraction does not.

We noticed that the proportion of cells in the density wave decreases smoothly over time—early in the 10 μM simulation it was 100%, but after 6 h of simulation, it was only about 25% (Fig 3B). This predicted decay in the fraction of the population that is chemotactic is confirmed by real cells in under-agarose assays (Fig 3E and 3F). Thus—counterintuitively—most of the cells are not chemotactic in a population that has been responding to a self-generated gradient for a significant time, even though the pattern of migration is completely defined by the chemoattractant.

To see what limits access to attractant for cells behind the density wave, we looked at mathematical models of this diffusion-degradation process (see text in S1 Fig for details). In two different models, we found that the number of cells needed to degrade all chemoattractant reaching them by diffusion decays with a very similar profile over time. We hypothesised that the density wave is composed of those cells that are exposed to detectable levels of attractant. These cells break down essentially all the attractant, so the cells behind cannot detect any at all. Cells behind the wave are therefore undirected, as they experience no attractant stimulus. This makes the system surprisingly robust. The wave contains exactly enough cells to break down all the attractant. If at any time there are too many cells in the wave, some are left behind. This means that the wave of directed cells behaves identically, irrespective of the size of the initial population. As the simulation includes no diversity in the properties of individual cells, these behaviours can be explained entirely by random differences in exposure to an external chemoattractant and require no phenotypic diversity.

One additional prediction of the computational model is that saturation delays the formation of the migratory wave but does not block it. At 100 μM attractant (more than three orders of magnitude above the K_*d*_ of the receptor), receptor saturation is overcome in simulations after a delay that allows cells to break down attractant to subsaturating levels and, therefore, to resolve a gradient and form a migratory front (Fig 3C). This waiting time effect is also replicated in live-cell experiments (Fig 3G, ~10^6^ cells in the well). This effect depends on degradation following Michaelis Menten kinetics, as otherwise, a 10-fold increase in concentration would simply result in a 10-fold increase in degradation rate, eliminating the waiting time.

The model also predicts that cells given an initial lead are likely to maintain it. In the simulated 10 μM attractant, cells that were found in the front quintile after 30 min were disproportionately more likely to be in the front quintile after 6 h (Fig 3G). This is not surprising; the initial leaders will remain exposed to the self-generated concentration gradient, and the resulting guidance will generally keep them in the lead; however, cells at the rear do not move directionally and are unlikely to catch up. This feature is replicated in the experimental data (Fig 3H, data tracked by hand for accuracy), though the experiment also contains a feature that is not predicted by the model—cells that start in the trailing quintile are mostly still consigned to the trailing quintile by the end of the observation time.

### Experimental Measurement of a Self-Generated Gradient

**Fig 5:**
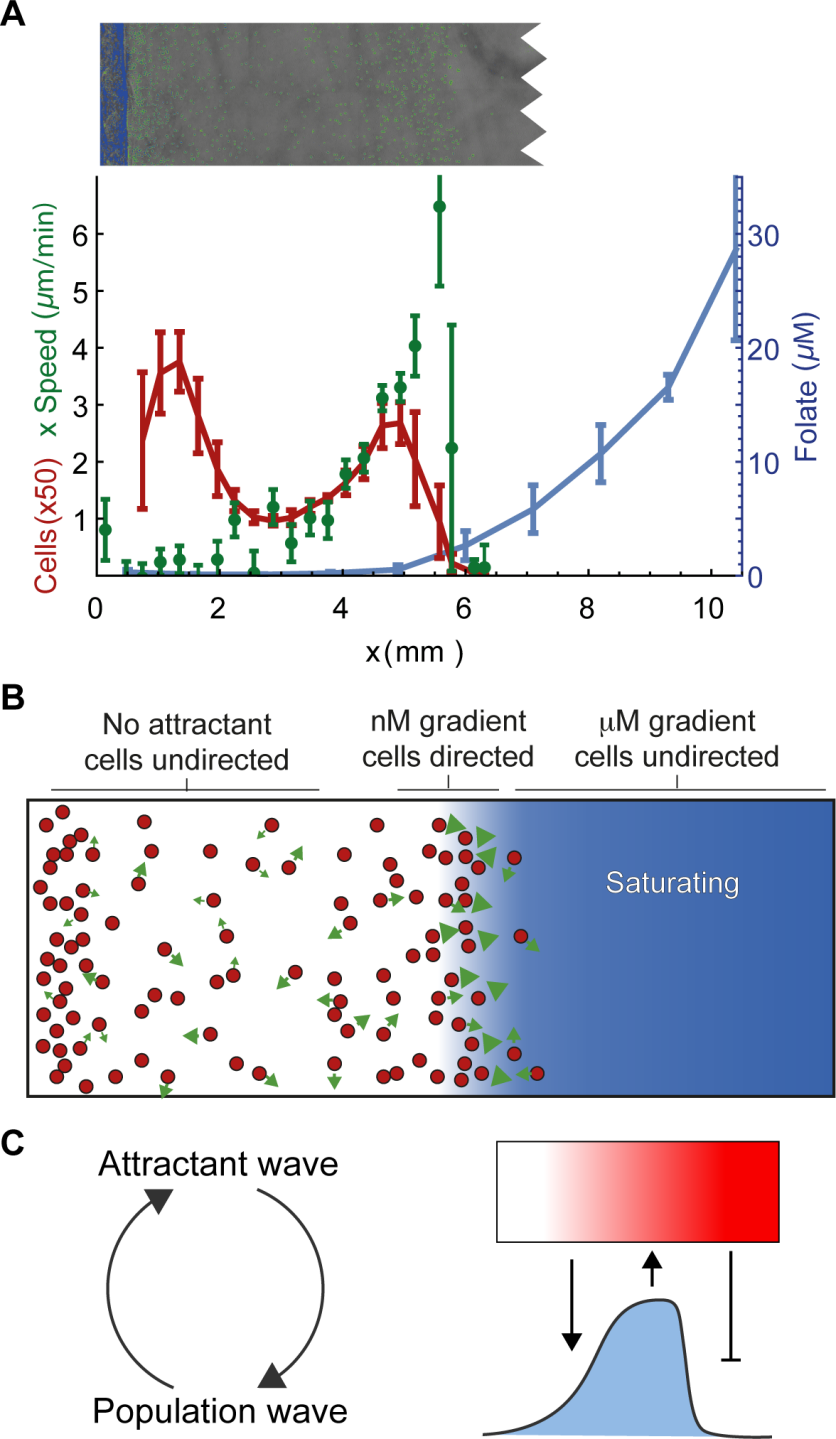
Mass spectrometry confirms model prediction of migratory wave as an attractant sink. (A) Distribution of population (red), average x component of velocity (the component directed away from the well) (green), and folate concentration, as determined by mass spectrometry. As predicted by the model, the folate concentration profile descends to a sink in the population wave. Overhead shows a microscope image of the assay from a single technical repeat aligned with the graph. (B) Diagram of the situation in A. Cells are distributed with a density peak toward the front. Attractant (blue) is metabolised by this density peak, leaving none (or almost none) behind it. Only the cells in the density peak are given strong chemotactic signals and migrate forward. (C) A model of mutual reinforcement by the attractant and density waves. The density wave shapes attractant by degradation, and the resulting environmental profile causes a central tendency in chemotaxing cells, maintaining the higher density region. doi: 10.5525/gla.researchdata.252.

Our computational models and microscope observations suggest that self-generated chemotactic gradients behave in a number of unexpected ways. Under most conditions, it would be extremely hard to confirm these by direct measurements. Self-generated gradients are by definition unstable, dynamically formed, and change rapidly in space and time. Fortunately, however, under-agarose assays are unusual—they are relatively robust, evolve slowly, and the attractants are both immobilized by the agarose and accessible. We therefore performed under-agarose chemotaxis assays with cells migrating towards 40 μM folate over 10 h. For each assay, we made phase-contrast movies of the last hour of the assay and used these to measure cell migration, chemotaxis, and the final position of each cell (Fig 5A). We then sliced the agarose above the cells into ~1 mm sections and measured the folate concentrations, using mass spectrometry, from the well to beyond the position of the front, calibrating with agarose samples containing known folate concentrations.

This experiment yielded results that clearly confirmed our models (Fig 5A). As predicted, folate concentration falls rapidly to a sink in the population density wave and is negligible behind this point. Though the agarose initially contained 40 μM folate, the concentration above the wave of cells is below 1 μM. The gradient of folate in front of the wave is consistent with degradation of all attractant by the cells in the wave. This finding gives us an exceptional confirmation of our simulation prediction and, by extension, our conceptual model (Fig 5B and 5C).

### The Effects of Other Mechanisms on the Migratory Wave

**Fig 6:**
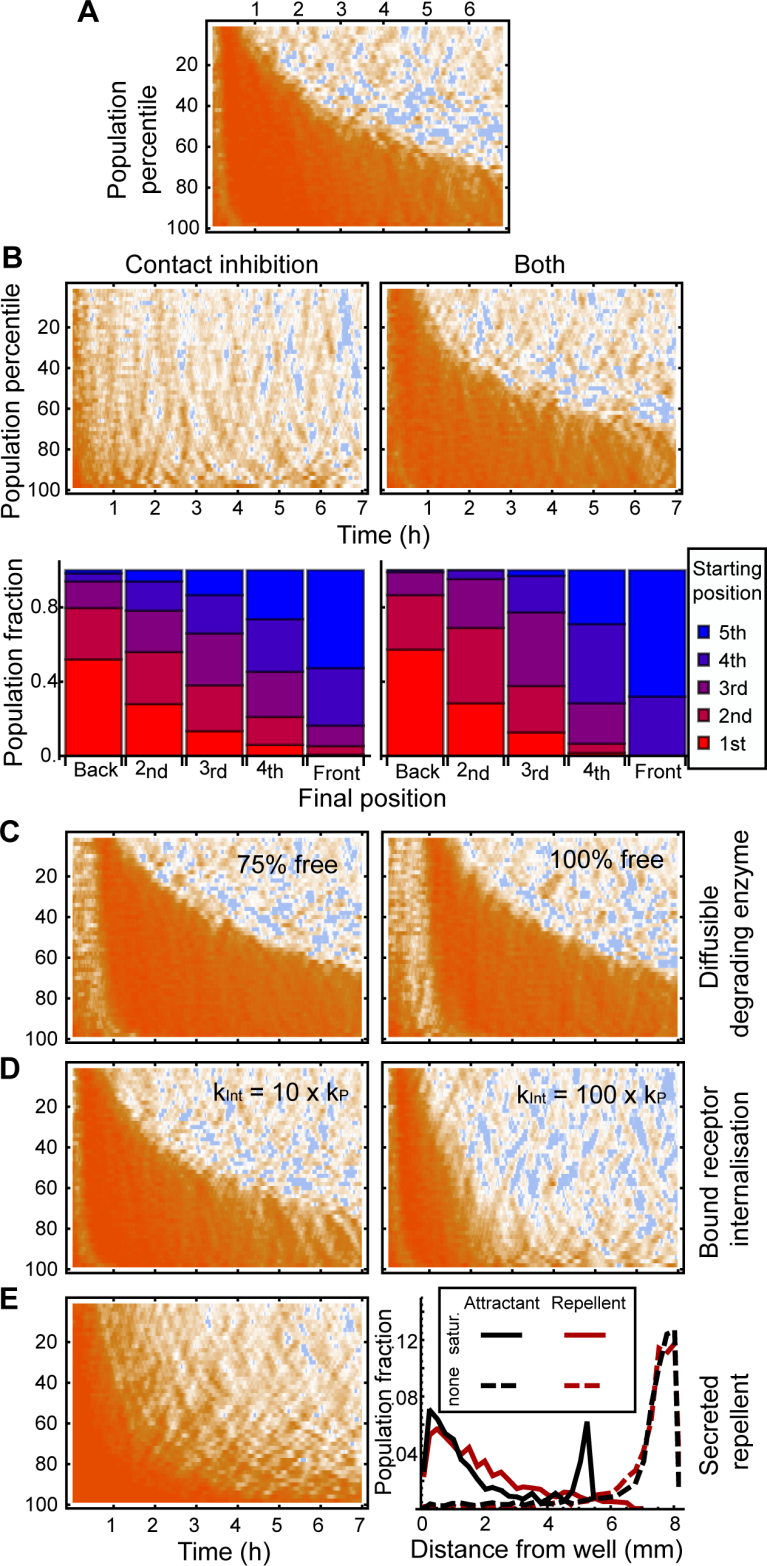
The population wave is diagnostic of a self-generated gradient. (A) Migratory dynamics of a typical 10 μM simulation. (B) Migratory dynamics for contact inhibition with and without attractant degradation. Graphs below show that CIL makes the additional prediction that cells that start near the well end near the well. (C) Inclusion of a diffusible degrading enzyme. Even where all degrader is free to diffuse, the migratory wave forms and decays. (D) Inclusion of receptor internalisation. (E) Migratory dynamics and population distribution at 7 h with secreted repellent. doi: 10.5525/gla.researchdata.252.

To test whether the leading wave of cells could be used to diagnose the presence of self-generated gradients, we compared our model results for a 10 μM self-generated gradient (Fig 6A) against cells steered by a variety of other mechanisms (of course each of these mechanisms is worthy of a study in itself, and so we explore them here only in sufficient depth to observe their influence on our principal findings).

We first examined whether contact inhibition of locomotion (CIL), a mechanism that has been strongly implicated in directional motility [17], could lead to the formation of a wave of migrating cells. Though this mechanism has not been unambiguously defined, we chose an effect that is consistent with the definition in Mayor [18] in which cells that make contact with one another are directly inhibited from moving in the direction of contact and thus migrate in a new direction (see supplementary material for details).

We first ran a simulation to see if contact inhibition-driven migration could also explain the exponentially decaying form of the dynamic leading front observed both in the data and in the self-generated gradient simulations (Fig 6B). Cells driven by contact inhibition alone did spread outwards from their original site but did not form the leading wave that formed using a self-generated gradient. This excludes contact inhibition as a principal driver of migration where the wave is observed.

We also constructed a mixed model driven by both contact inhibition and a self-generated gradient of attractant. This showed a clear migratory wave like the simple self-generated model, indicating that the wave is diagnostic of self-generated gradients, even where additional mechanisms are acting (Fig 6B, mixed model).

The leading quintile of the early population is predicted to maintain its lead when driven by contact inhibition, as it is in self-generated gradients as discussed earlier. However, the model of contact inhibition also shows a disproportionate number of the cells that start in the trailing quintile to finish in the trailing quintile, where no strong prediction is made by the self-generated gradient model. This is true both for contact inhibition alone and in the case of the mixed model. We therefore suggest that this failure of the rearmost cells to mix is a feature that can positively indicate the action of contact inhibition. Note that it requires sufficient population density to generate frequent contact effects, and so the effect may not always be present, even in cells that can be steered by contact inhibition (though it certainly is where contact inhibition has any major role). The experimental data described earlier include features particular to both of these mechanisms, which cannot be explained by either alone. We therefore suggest that a strong model for the spreading assay is in fact a mixed model of CIL and degradation.

Our initial simulations assumed that all degradation happened locally to the cell, meaning that all degrading enzyme is bound to the cell surface. As both cAMP phosphodiesterase and folate deaminase are also secreted into the environment, we tested whether a freely diffusing degrading enzyme would also yield a travelling wave. We ran simulations in which most and all degrader was free to diffuse in the environment, rather than remaining bound to the cell (Fig 6C). We assumed that attractant degradation rate was proportional to the local degrader concentration, and that the Michaelis-Menten constant remained the same as for the bound degrader. As folate deaminase is a large molecule (MW ~40,000, [13]), we chose a diffusion coefficient that was small relative to that of folate (70 μm^2^/s). One clear difference was a small lag time before the wave forms, due to the time taken for degrader to accumulate. After this, it behaved very similarly to the simulations that use only bound degrader. The principal findings remain unaffected, however, as even with all degrader free to diffuse, the wave quickly formed and followed the same decaying pattern.

We next explored the potential effects of receptor internalisation (Fig 6D). We included a simple model of the process, in which naive receptor production and degradation rates at equilibrium result in the same chemotactic sensitivity as in the base simulation. Exposure to attractant causes more rapid internalisation, lowering the equilibrium sensitivity. As the self-generated gradient mechanism always lowers the concentration experienced by the wave to nanomolar levels, it is remarkably robust to increases in turnover rates. We found that, even for rapid bound-receptor internalisation (10x the production rate), we still observed the formation and decay of the wave. Rapid internalisation can eventually be made to break the wave—at 100x the production rate, the wave forms but is quickly unable to resolve a gradient strong enough for the low sensitivity that cells have at equilibrium and so collapses. This extreme case does not resemble the experimental data, however, so it cannot be considered an appropriate model for our system.

We finally considered a secreted repellent. We simulated chemoreception and chemotaxis with identical dynamics to the attractant, but with the sign of the bias reversed. We present the case where secreted repellent diffused with a diffusion coefficient of 30 μm^2^/s. This mechanism does recapitulate elements of the self-generated gradient behaviour: the front of the population moves in a definite, directed fashion over a similarly long distance, and a decreasing proportion doing so accurately, as large concentrations of repellent diffusively overtake them and saturate their receptors. A key difference is that the front is not well defined, with cells toward the rear gradually performing worse chemotaxis. We attribute this difference to the fact that saturation by repellent is gradual, whereas attractant depletion results in a more abrupt cut-off where the concentration reaches zero. We also observe no density peak in these simulations (compare attractant-driven population distribution, Fig 6E, solid black line with repellent-driven distribution, solid red line). We suspected that this resulted from a reversal in the effects of saturation: in the case of an attractant, the receptors of the cells at the very front of the wave saturate, causing them to drop back, whereas those cells at the rear experience a relatively good signal and so catch up (at least, until attractant depletion is essentially total). In contrast, it is the rear of a repellent-driven migration that experiences more saturation and thus poorer directional resolution. We tested this by comparing two simulations with no saturation mechanisms, one based on repellent secretion and the other on attractant depletion (Fig 6E, dashed lines), and found that, once saturation effects were removed, these conditions were equivalent.

### The Effects of Degradation on Externally Generated Chemoattractant Gradients

Previous work [14] and the data presented above clearly show that cells can create local gradients from homogeneous surroundings. This made us ask whether degradation is also important in more usual chemotaxis assays, in which attractants are applied as gradients (for example [19]). In chemotaxis chamber assays, a bridge separates wells containing an attractant source and a sink; fluorescent tracers show that a linear gradient forms across the bridge. Most authors presume that the attractant is also linear, but this neglects the effects of the cells degrading the attractant.

**Fig 7:**
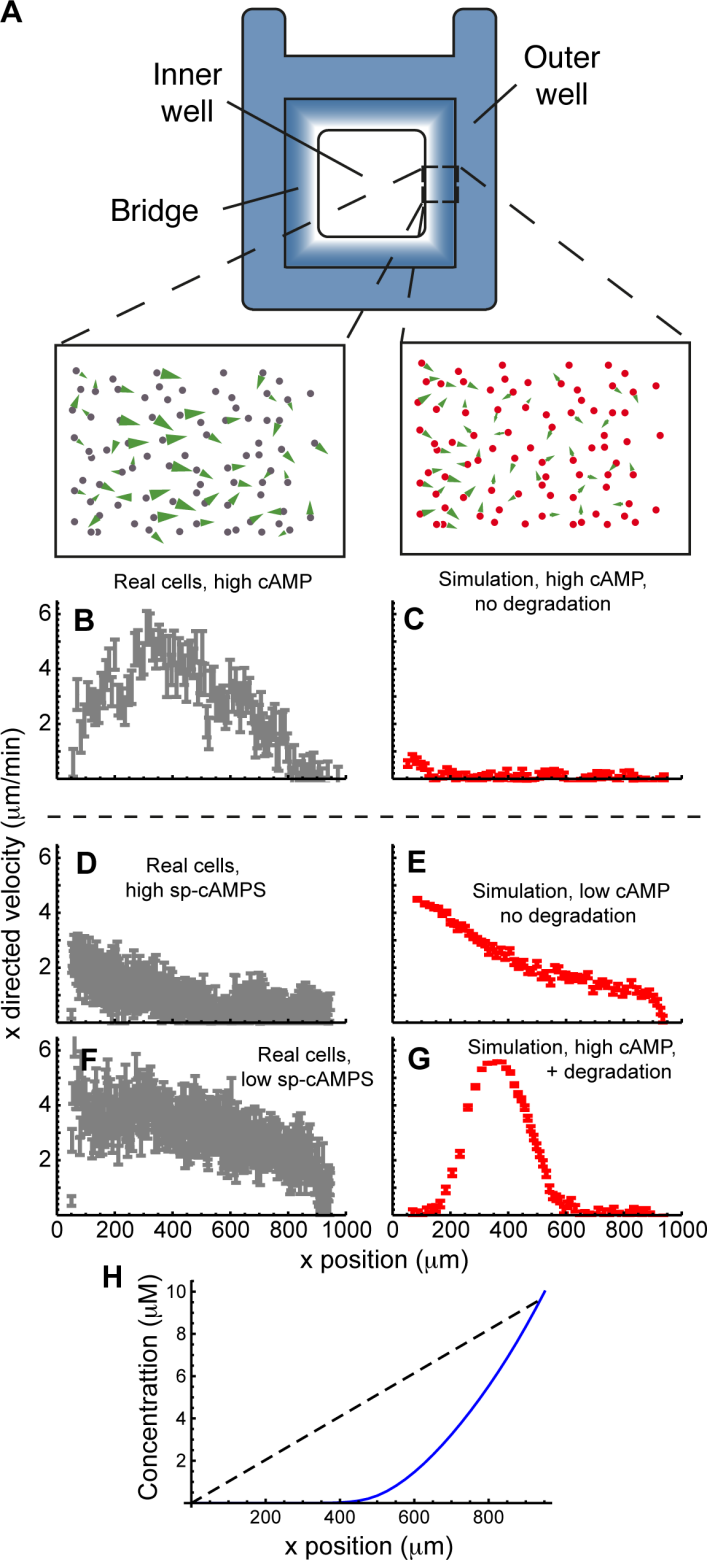
Degradation is important to chamber assays. (A) Diagram of the experiment. Cells are evenly distributed in a chamber with an outer well containing attractant and an inner well containing attractant-free medium. In the absence of external influences, this will create by diffusion a linear gradient of the attractant leading towards the outer well. We observe the migratory behaviour of cells on a bridge between these two wells and graph the profile of velocity toward the outer well. Cartoons of this behaviour are shown above (B) and (C), with cell movement shown by green arrows. (B, D, and F) Velocity in the direction of the imposed gradient for *Dictyostelium* cells in an Insall chamber for (B) 10 μM cAMP, (D) 200 μM Sp-cAMPS and (F) 600 nM Sp-cAMPS. (C, E, and G) Velocity of cells in a simulated Insall chamber-like condition, with (C) high cAMP and no degradation, (E) low cAMP and no degradation, and (G) high cAMP with degradation. (H) Gradient predicted by the simulations with (blue) and without (black, dashed) degradation. This shows how much degradation can cause the profile to deviate from the originally imposed linear gradient. doi: 10.5525/gla.researchdata.252.

We first simulated a chemotaxis chamber assay under typical conditions, allowing no degradation of chemoattractant by cells and assuming an established gradient from the start. We used a gradient of 0–10 μM across 1 mm in an Insall chamber (Fig 7A), which for real cells yields strong chemotaxis across the chamber (Fig 7B) with a peak at the centre of the bridge. However, in the absence of degradation, nearly all simulated cells were saturated by these levels of chemoattractant and were therefore unable to chemotax (Fig 7C). Only those cells at the extreme low end of the concentration gradient were able to resolve and respond to the directional signal.

To test whether this extreme difference between modelled and real conditions was simply due to attractant degradation, we tested the effect of attractants that could not be degraded. We used Sp-cAMPS, a nondegradable analogue for cAMP [20], at concentrations that give equivalent receptor occupancy (the affinity of the receptor for Sp-cAMPS is weaker than for cAMP)[20]. This ensured that the cells would experience a linear attractant gradient. In this case, we observed a similar response to that predicted by the simulation (Fig 7D). Most cells were not chemotactic, with only cells at the low end of the concentration gradient able to chemotax at all. Thus, the difference between simulated and real chemotaxis assays can be fully explained by chemoattractant degradation.

As saturation effects were so profound in the simulated condition above, we tested whether lower chemoattractant concentration would overcome this issue and result in good chemotaxis. We repeated the simulation and the real experiment using maximum concentrations only slightly in excess of the K_*d*_ of the receptors (Fig 7E and 7F). The simulation predicted positive chemotaxis across the whole domain, with a steady decline in the x-component of velocity as the background attractant concentration increased and the relative difference across the cells dropped. This prediction was recapitulated with remarkable accuracy by the experimental results. Thus the lack of chemotactic response in the high-Sp-cAMPS assay was caused by receptor saturation.

These results made it seem likely that the widely-used assay using 10 μM cAMP absolutely depends on cAMP degradation to be able to function. We therefore repeated the simulation shown in Fig 7B, with a 10 μM cAMP source, but this time allowed local degradation of chemoattractant. The simulation predicted excellent chemotaxis (Fig 7G), with a peak of directed movement in the middle of the chamber, exactly as seen with real cells. Cells either side of this peak are less chemotactic for opposite reasons: those close to the source are exposed to too much chemoattractant and are saturated, as too few cells have contributed to degradation to bring the attractant levels into a resolvable concentration range. Those on the sink side are not exposed to sufficient attractant, as it has largely been degraded by cells closer to the source. The simulations reveal that the gradient to which cells respond is not at all the linear gradient that we aim to impose. Without degradation, the gradient is indeed linear (Fig 7H, black dashed line); however, when degradation is allowed to occur, the resulting gradient is clearly nonlinear, being better matched by a decaying exponential (Fig 7H, blue line).

The accuracy of the model that includes attractant degradation leads to three surprising conclusions:

1. in assays using Zigmond, Dunn, and Insall chambers [15,21,22], attractant degradation is extremely important in generating the final gradient profile perceived by the cells;
2. chemoattractant degradation not only shapes the gradient, but massively changes the absolute attractant levels—the midpoint in the simulated gradient changes from a saturating 5 μM cAMP to below 1 μM, and in the lower half of the gradient, the limitation on chemotaxis changes from receptor saturation to insufficient attractant;
3. linear gradients—widely discussed in the literature—are unlikely to exist under conditions where the attractant can be broken down. Similarly, fluorescent tracers completely misrepresent attractant gradients under these conditions.

### Externally Imposed Gradients and Self-Generated Gradients Are Useful under Different Conditions

**Fig 8:**
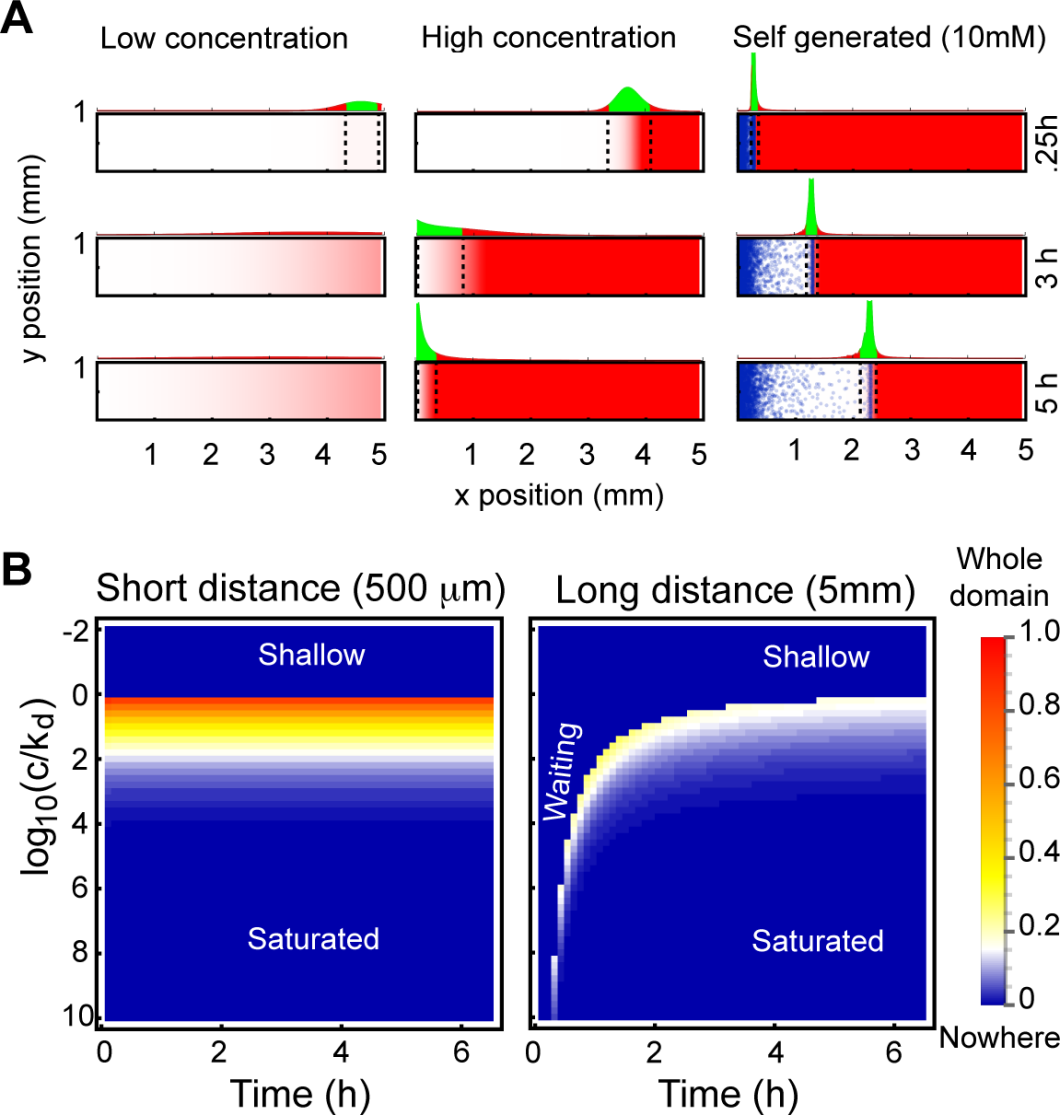
Limitations of guidance by point sources. (A) Chemotactic regions for a localised source of attractant. Assuming chemotaxis requires a 1% relative occupancy difference, we can indicate the regions where a directional signal can be resolved (however poorly). Each panel shows a snapshot of a simulation of attractant diffusion from a point source at the right-hand side. Dotted lines bound the region in which chemotaxis can occur. Graphs above each simulation show receptor occupancy difference, with regions of ≥1% occupancy difference filled in green, and regions of <1% filled in red. The leftmost column shows time points for a source of low concentration. The middle column shows a source of high concentration. The right-hand side shows a self-generated gradient, in which the area in which chemotaxis can occur follows the density wave as it travels. (B) The fraction of the region in which chemotaxis can occur for short- and long-range point source, point-sink systems in the absence of degradation, displayed as a function of source concentration (on the *y*-axis) and time given to diffuse (on the *x*-axis). Hotter colours indicate better coverage of the region, with red indicating guidance over the entire domain and blue indicating no guidance. Note that this colour does not reflect on the strength of the guiding cues, only on their extent in space. The graph assumes a diffusion coefficient of 300 μm^2^/s. doi: 10.5525/gla.researchdata.252.

The results we have described highlight a fundamental problem with externally imposed gradients, in which a local source diffuses to create a chemotactic gradient. Shallow gradients, generated by low source concentrations, are too flat for cells to resolve; steep gradients, generated by high source concentrations, rapidly lead to saturation as cells climb the gradient. Furthermore, diffusion is slow over longer distances. The distance travelled by a diffusing molecule is proportional to the square root of time, so as distances increase, it takes an impractical time to establish a resolvable directional signal (Fig 8A). These different parameters interact. A signal can always travel further faster by increasing the source concentration, but this restricts the domain of guidance by making more proximal areas saturating and therefore useless for directional guidance.

Self-generated gradients are far less limited by these effects. Because gradients are highly localised, relatively low concentrations of attractant can be shaped into perceptibly steep gradients. Conversely, when concentrations of attractant are very high, the cells remain stationary for long enough that the attractant is locally broken down to subsaturating levels before they start to move (see Fig 3).

Conversely, while externally-applied gradients can inform all of the cells within their field, self-generated gradients instruct only that proportion of the cells that are within the front wave. Thus only a subset of cells is steered at any time, and the number of cells in the wave decreases as the wave moves.

To compare the usefulness of externally-imposed and self-generated gradients as guidance cues are over different distances, we calculated the fraction of the domain over which guidance was plausible (that is, the fraction of the area between the source and sink in which chemotaxis is possible—see S1 Fig for details). In performing this calculation, we assume that a relative difference of 1% receptor occupancy is required for guidance, and that a minimum overall occupancy of 1% of all receptors is also required; both assumptions are physiologically plausible (Fig 8B). At short distances (below 1 mm), we find that guidance can be achieved over the whole domain for source concentrations near and above the K_*d*_ of the receptor. Lower source concentrations never yield steep enough gradients to create a 1% occupancy difference, whereas source concentrations that are considerably higher create increasing saturation effects, diminishing the region of effective guidance.

Over longer distances, both the shallow region and the saturating region can again be seen, with an additional “waiting” region, in which cells distal from the source are still waiting for appropriate guidance cues to reach them. Over these distances, even the best combinations of waiting time and source concentration result in only moderately sized regions of guidance. This indicates that simple gradients are a severely limited guidance cue at distances greater than 1 mm. However, self-generated chemotaxis can act over arbitrarily large distances and times, because the gradient is local and moves through the field. Thus, for fields that are smaller than 1 mm, externally-generated gradients such as point sources will often be the most efficient mechanism for steering cells, but above 1 mm, self-generated chemotaxis will be increasingly advantageous. It is likely that most or all instances of chemotaxis over a greater distance than 1 mm involve a self-generated gradient, rather than a local source alone.

## Discussion

It is obvious that cells can create gradients by breaking down attractants locally. Many different scenarios in which this occurs have been described [23–27], and its importance at the single-cell level has been investigated theoretically [28]. However, there are many significant and unexpected differences between such self-generated gradients and “typical” chemotactic gradients, in which external factors impose the gradient and the chemotactic cells simply respond to it. In particular, the self-generated gradients work relatively better as the range (that is, the distance over which migration is chemically guided) gets larger. Over short distances (up to 1 mm, as shown in Fig 8, externally-imposed gradients are effective, but at longer ranges they work dramatically less well, whereas self-generated gradients are effective over an arbitrarily large range. The behaviour of different cells within the population is also fundamentally different. In externally-imposed gradients, all cells respond similarly, whereas in self-generated gradients, only the cells at the front of the population are guided. Cells at the rear of the population do not perceive a chemotactic gradient and are not directed. This is counterintuitive—even though the behaviour of the population is entirely determined by the chemoattractants and the gradients they form, most of the individuals in that population do not experience a gradient. Also counterintuitive is the observation that a constantly diminishing fraction of the population of cells responding to a self-generated gradient is in fact steered by the gradient. All of these observations conflict with the chemotaxis field’s view of a “typical” chemotactic response, which is shaped by tests like Zigmond chamber and transwell assays, in which the experimental conditions are tuned to give a relatively consistent result from cell to cell. The problem for biologist’s intuition is that the chemotactic behaviour of a single cell does not scale simply when examining the responses of a population.

Even in common experimental systems (the “typical” responses described above), cells are likely actually exposed to self-generated gradients, derived from the pattern of added attractants but greatly altered by the cells. As our Fig 7 shows, the behaviour observed in chemotaxis assays is not consistent with the concentrations of attractants that have been imposed—the real attractant levels perceived by the cells appear to be orders of magnitude lower. Given our findings about the limitations of external gradients—small ranges and relatively narrow dose-responses—we believe self-generated chemotaxis is more widespread in biology than is at present realised. Detailed reanalysis of known chemotaxis systems in medicine and biology will probably reveal some that are fully self-generated, some that incorporate elements of self-generation, and others that involve simple chemotaxis towards agents that are secreted and broken down by other cells. Our descriptions of the behavioural traits that emerge from self-generated chemotaxis (in particular the two states of behaviour and the decay of the leading edge) may be used to detect it in other systems that are hard to interrogate directly.

There is little recognition of the role of ligand breakdown in changing the shape or dynamics of chemoattractant gradients, and there are only a few previous observations of physiological self-generated chemotaxis. This is perhaps not surprising, because the gradients are exceptionally hard to visualise. Several authors have used fluorescent molecules of similar size to attractants, to act as markers in chemotaxis assays. Such markers may reproduce the effects of external sources and sinks but of course cannot reveal the effects of attractant degradation on the evolution of the gradient. Diffusible markers have led to the conclusion that chemotaxis chambers can produce linear gradients (in comparison to microneedles, which generate exponentially decaying gradients), but our work shows this is only true if the cells do not substantially break down the attractant. Our data show the "linear" gradients in Zigmond, Dunn, and related chambers [15,21,22] are most likely converted to exponential gradients by the responding cells (Fig 7H). This should, in hindsight, have been obvious. The concentrations of attractant that give the best chemotactic responses are nearly always far higher than the optimal response range for the cells, and chemoattractant degradation has been known for some time. In chambers, even with substantial attractant reservoirs acting as a source and sink, gradients are substantially shaped by the same process that makes self-generated gradients. This is a great surprise—there is a large body of literature on the "linear gradients" in such chambers, in which the cells are typically thought to be responding to on average 50% of the attractant concentration in the source; we have shown that it is usually far less than that, and that the gradients are far from linear. This emphasises the complexity of chemotactic mechanisms [29]. Future quantitative studies must clearly pay attention to breakdown as well as diffusion if they are to understand the key parameters of chemotaxis.

The difficulty in visualising attractant breakdown will be an ongoing problem for the field. Fluorescent tracers have revolutionized much of cell biology, but in this context are only useful if the tracer’s fluorescence is clearly altered at the same time as it is enzymatically broken down. The fluorescence of markers such as mant-cAMP and fluorescently-tagged LPA will not be altered upon attractant breakdown, so they are useless for measuring self-generated gradients. Both LPA and cAMP are broken down by phosphodiesterases, but hydrolysis-sensitive fluorescent modifications around the active phosphates will obviously affect the ability of the molecules to interact with both receptors and enzymes. In a technical tour de force, Tomchik [30] directly measured waves of chemoattractant in *Dictyostelium*—the focus in this case was on the generation of the waves, but the approach could work for self-generated gradients. A number of recent approaches have indirectly measured ligands through their effects on receptors. Dona et al. [7] used a “fluorescent timer,” in which the rate of receptor activation was measured through their turnover rate, to measure chemokine gradients in the zebrafish lateral line precursor. A similar gradient could also be measured using a ratiometric probe for receptor degradation [8]. These assays may allow other self-generated gradients to be measured in the future, but they are technically difficult and hard to validate. We have measured LPA gradients in melanomas in vivo [3], but with very poor resolution. Direct measurement of self-generated gradients would be very desirable in the future.

In cases where direct observation of gradients is not yet possible, the problem nonetheless remains tractable to computational investigation. As we have shown here (and elsewhere [31,32]), well-verified computational models are excellent scientific tools that can make accurate, nontrivial, and counterintuitive predictions about the system. Here, we have used them both to expand our understanding of the system’s dynamics and to guide the study, informing us which experiments are key to understanding the system.

Overall, however, this study offers a positive message—self-generated gradients are likely to be widespread in medicine, development and many other fields. The work described here suggests several different ways they can be inferred. They are as follows:

1. The most visually obvious sign for a self-generated gradient (though it does not occur clearly in all cases) is the leading wave of cells.
2. However, even in the absence of a leading wave of cells, self-generated chemotaxis may be inferred if the front of a population is strongly guided, but cells behind the front move randomly.
3. The near-exponential decrease over time of the proportion of responding cells seen in our simulations and measurements is also diagnostic of self-generated chemotaxis.
4. One key pointer—which has often been seen in the literature—is when the quantity of an attractant used in physiological conditions or in an assay is far in excess of the sensitivity range of the receptor. For example (see Fig 5A), under-agar chemotaxis assays with *Dictyostelium* use folate concentrations in the tens of micromolar, even though the K_*d*_ of the receptor is in the nanomolar range.
5. Self-generated gradients work over distances that are not possible for chemotaxis to an externally-imposed linear gradient.
6. A self-generated gradient is implied if chemotaxis happens in uniformly imposed attractant.
7. If a specific attractant-degrading enzyme can be found to be essential, or if normal and nondegradable attractants cause markedly different outcomes (see Fig 7), a self-generated gradient may be concluded directly.

For each of these tests, externally-imposed chemotaxis gradients give completely opposed results, while mechanisms like contact inhibition give related but clearly different outcomes. When observing cells in a system where the driving mechanism is unknown, it is hard to distinguish self-generated gradients formed by breakdown of an attractant [3] from those created by secretion of a chemorepellent [33]; as we show in Fig 6E, the behaviour of the population is similar enough that points 2, 3, and 5 may be difficult to tease apart by in vivo observation. As such, discriminating between these systems will likely require testing of conditioned medium for attractants and repellents, or direct identification of the players. For point 6, uniformly applied attractant could change migration by causing chemokinesis as well as via a self-generated gradient. In this case, all individuals of the population would behave similarly, so we would use points 1–3 to identify a true self-generated gradient.

Other tests will no doubt emerge. We foresee that applying such tests to physiologically important situations, and a more general understanding of this mode of chemotaxis, will lead to strong gains in our understanding of normal physiology and disease processes in general.

## Materials and Methods

Experiments were performed with AX3 *Dictyostelium discoideum*. Cells in Fig 1 were grown on bacteria. For all other figures, cells were grown in HL5 medium. Under-agarose assays were performed using 4 mL of 0.4% agarose in a 50 mm glass-bottomed dish, pretreated with BSA. Insall chamber assays [34] used cells starved on agar until the onset of streaming and then placed in buffer containing 2 mM caffeine to prevent cAMP release. Image capture was performed at 10x with a Qimaging Retiga camera. Tracking was performed automatically using a custom-written plug-in for ImageJ, in which candidate cells were found at minima in each frame of a Gaussian-filtered copy of the movie, with cells tracked from frame to frame by minimising the overall sum of square distances travelled. All primary data (and recorded cell tracks) are available via the DOI: http://dx.doi.org/10.5525/gla.researchdata.252.

Simulations were agent-based, with each cell deciding on its direction of motion according to local cues. Each cell performed a persistent, biased random walk in 2-D. Step distance was the same for each iteration. The direction of each step was determined by a circular average of the direction of chemotactic bias, weighted according to its strength, and a persistent random step drawn from a wrapped normal distribution, centred on the current direction of motion. The chemotactic bias was determined by receptor occupancy difference across the cell, with receptors following standard bimolecular equilibrium behaviour, except where stated otherwise. Quantifications performed on simulated under-agarose assays ignored cells still in the first 900 μm of the simulation to provide space for a well-like region in which cells interact with the attractant, but are not under observation.

Chemoattractant concentrations and gradients were interpolated from a grid of stored values, with these values updated by simulating diffusion using a central differences scheme. Attractant degradation was simulated by removing attractant from the grid point most proximal to the cell, and the rate of depletion followed Michaelis-Menten kinetics. All simulation outputs are available via the DOI listed above, and source code is available at: https://github.com/ltweedy/SGG_Simulation.

## Supporting Information

S1 Fig Details of the mathematical models used to understand the decay of the travelling wave population in Fig 3 and to demonstrate the limitations of static, linear gradients in Fig 8. (PDF)

S1 Movie Long-range self-generated chemotaxis of *Dictyostelium* cells towards folate. (MOV)

S2 Movie Agent-based computational simulation of self-generated chemotaxis. (MOV)

S3 Movie Explanation of heat maps shown in Fig 3. (MOV)

S4 Movie “Bubble” processed movie showing directional migration within a self-generated population wave. (MOV)

## Abbreviations

CIL: contact inhibition of locomotion
LPA: lysophosphatidic acid
