# Supplementary material for: Self-Generated Chemoattractant Gradients: Attractant Depletion Extends the Range and Robustness of Chemotaxis
Source: PLoS Biol. 2016 Mar 16;14(3):e1002404. doi: 10.1371/journal.pbio.1002404 (PMC4794234; doi:10.1371/journal.pbio.1002404)
Supplement: S1 Fig — (PDF) [file pbio.1002404.s001.pdf]

Supplementary Information for Self-Generated Chemoattractant Gradients: Attractant Depletion Extends the Range and Robustness of Chemotaxis

Luke Tweedy, David A. Knecht, Gillian M. Mackay and Robert H. Insall

1 Supplementary materials and methods

1.1 Experimental methods

Chemotaxis toward folate was investigated using the one-well under-agarose assay. A 50mm glass-bottomed dish is pre-treated with 10mg/ml BSA in phosphate buffer. Attractant is mixed into 0.4% agarose and is allowed to set in the dish. A long thin well (20mmx2.5mm) is cut from the agarose in the middle of the dish, and 100μl of a $2x10^6$cells/ml suspension is added. Imaging was performed in phase contrast using a 10x objective lens, and focused on cells as they move under the agarose layer.

Chemotaxis assays toward cAMP and Sp-cAMPS were performed in an Insall chamber. AX3 cells were developed on 1.5% agar until streaming became visible. These cells were harvested in development buffer (DB) and placed into an Insall chamber. The outer well was filled with DB containing attractant, the inner well with attractant-free DB. Media in both wells contained 2mM caffeine to prevent cAMP release by cells.

1.2 Computational model

The model consists of a diffusible attractant and a population of cells at discrete locations, able to degrade attractant locally. The profile of attractant concentration $c(\mathbf{x}, t)$ changes as follows:

$$\partial_t c(\mathbf{x}, t) = D_c \nabla^2 c(\mathbf{x}, t) - \sum_{n=1}^{N} r(c(\mathbf{x}_{n,t}, t))\delta(\mathbf{x}, \mathbf{x}_{n,t}) \tag{1}$$

Here the first term represents the diffusion of the attractant, with a diffusion constant D_c. The second term sums the contributions of a population of N cells to the degradation of attractant, with the nth cell found at the location $\mathbf{x}_{n,t}$ at time t. Each cell degrades

attractant explicitly locally (enforced here by the Kronicker delta) and at a rate r, which depends on the local concentration. r takes the form:

$$r(c) = \frac{r_{\max}\, c}{c + k_M}, \tag{2}$$

where $r_{\max}$ is the rate of decay when the cell is saturated with attractant, and k_M is the Michaelis-Menten constant.

Practically, diffusion of attractant in the model was simulated on a grid using a central differences approximation of the diffusion equation. The degradation term was simulated using the Euler method. Cells do not move on the diffusion grid, and so can take intermediary positions. As such, degraded attractant was taken from surrounding grid points, linearly interpolating the rate based on their distances from the cell. In simulations of the Insall chamber, the boundary conditions $c(0, y, t) = 0$ and $c(L, y, t) = c_{\max}$ are applied, approximating the effects of the large resevoirs of medium at either end of the bridge.

Cells in simulations of the one-well assay begin in uniformly-distributed random positions in a thin (500μm) band along the left-hand side of the simulation. They move from their positions at a constant speed in a persistent, biased random walk. In each time step of size Δt, the nth cell chooses a direction $\theta_{n,\,t}$ in which it will move in the interval $(t,\ t + \Delta t]$, by taking a weighted circular average of a persistent random walk $\mathbf{p}_{n,\,t}$ and a bias introduced by local chemical cues $\mathbf{b}_{n,\,t}(c(\mathbf{x}, t), \mathbf{x}_{n,\,t})$:

$$\theta_{n,\,t} = \arctan\left(\frac{(p_{n,\,y,\,t} + k_b b_{n,\,y,\,t}(c(\mathbf{x}, t), \mathbf{x}_{n,\,t})}{(p_{n,\,x,\,t} + k_b b_{n,\,x,\,t}(c(\mathbf{x}, t), \mathbf{x}_{n,\,t})}\right). \tag{3}$$

The direction of the persistent, random contribution to the cell's movement is a random variable chosen from a wrapped normal distribution, centred on the previous direction of motion $\theta_{n,\,t-\Delta t}$.

$$\mathbf{p}_{n,\,t} = \cos(\Theta_{WN}(\theta_{n,\,t-\Delta t}, \sigma\sqrt{\Delta t}))\hat{\imath} + \sin(\Theta_{WN}(\theta_{n,\,t-\Delta t}, \sigma\sqrt{\Delta t}))\hat{\jmath} \tag{4}$$

where σ controls the strength of the persistence, and can be seen as the standard deviation in direction per unit time. For the bias $\mathbf{b}_{n,\,t}(c(\mathbf{x}, t), \mathbf{x}_{n,\,t})$ we choose the difference in the probability of occupancy for a receptor between the very front and back of the cell. Assuming the cell is projected across some length l:

$$\mathbf{b}_n(c(\mathbf{x}), \mathbf{x}_n) = \frac{c(\mathbf{x}_n + l/2)}{c(\mathbf{x}_n + l/2) + k_d} - \frac{c(\mathbf{x}_n - l/2)}{c(\mathbf{x}_n - l2) + k_d}. \tag{5}$$

The change in the position of the cell over the period $(t, t + \Delta t]$ is then

$$\Delta\mathbf{x}_{n,\,t} = v_c\big(\cos(\theta_{n,\,t})\hat{\imath} + \sin(\theta_{n,\,t})\hat{\jmath}\big)\Delta t. \tag{6}$$

Contact inhibition of locomotion is defined as occuring when two cells come into contact, repolarise and move in a new direction. We simulate this by giving each cell a region of

occupancy, and testing other cells for contact when they move. If a contact is detected, the component of movement in the direction of the contacted cell is removed. That is, for cell m, which has made contact with cell n, the new update in position $\Delta \mathbf{x}^*_m$ is

$$\Delta \mathbf{x}^*_m = \Delta \mathbf{x}_m - \frac{(\mathbf{x}_n - \mathbf{x}_m).\Delta \mathbf{x}_m}{|(\mathbf{x}_n - \mathbf{x}_m)|} \tag{7}$$

2 Mathematical models of the population density wave

2.1 Population density wave in a semi-unbounded domain

Let us consider a concentration distribution of attractant $c(x,t)$, initially evenly distributed at a concentration c_0 for all $x > 0$ which evolves according to the diffusion equation:

$$\partial_t c(x,t) = D_c \partial_{xx} c(x,t). \tag{8}$$

This attractant is degraded rapidly by a population of cells, which act as a perfect sink. These cells begin in a well at $x = 0$. As the well is long and the cells evenly distributed, we can treat this as a one dimensional problem. Let the population of cells move over time to a position $s(t)$, which is reached by simple chemotaxis, advancing proportionally to the local gradient:

$$\partial_t s(t) = \frac{k}{c_0} \partial_x c(s(t), t). \tag{9}$$

Eqn. (8) is satisfied by the function

$$\begin{aligned}
c(x,t) &= A\left(\operatorname{erf} \frac{x}{\sqrt{4D_c t}} - \operatorname{erf} \alpha\right) && (10)\\
\partial_t c(x,t) &= -\frac{Ax}{\sqrt{4\pi D_c t^3}} \exp \frac{-x^2}{4D_c t} && (11)\\
\partial_x c(x,t) &= \frac{A}{\sqrt{\pi D_c t}} \exp \frac{-x^2}{4D_c t} && (12)\\
\partial_{xx} c(x,t) &= -\frac{Ax}{\sqrt{4\pi D_c^3 t^3}} \exp \frac{-x^2}{4D_c t} && (13)
\end{aligned}$$

where $\operatorname{erf} x$ denotes the error function of x. As $c(x,0) = c_0,\ x > 0$

$$\begin{aligned}
A &= c_0/(1 - \operatorname{erf} \alpha)\\
&= c_0/\operatorname{erfc} \alpha, && (14)
\end{aligned}$$

where $\operatorname{erfc} x$ is the complementary error function of x. Substituting this into Eqn (10) gives us

$$c(x,t) = \frac{c_0}{\operatorname{erfc} \alpha}\left(\operatorname{erf} \frac{x}{\sqrt{4D_c t}} - \operatorname{erf} \alpha\right) \tag{15}$$

As the wave at $s(t)$ is a perfect sink, we know that $c(s(t),t) = 0$. Thus,

$$\begin{aligned} \operatorname{erf}\frac{s(t)}{\sqrt{4D_c t}} &= \operatorname{erf}\alpha && (16) \\ s(t) &= \alpha\sqrt{4D_c t} && (17) \\ \partial_t s(t) &= \alpha\sqrt{\frac{D_c}{t}} && (18) \end{aligned}$$

We can use Eqns (17, 18) to estimate the value of α for our system from the behaviour of the wave. Fitting the mean x position of forward-moving cells over time, we estimate that $\alpha = 1.23$. We can use Eqns. (9) and (12) to show that:

$$\sqrt{\pi}\alpha \mathrm{e}^{\alpha^2} \operatorname{erfc}\alpha = \frac{k}{D_c}, \qquad (19)$$

and so can use our estimate of α to calculate a value for k.

Though the wave moves over time, attractant only reaches the wave over minuscule length scales by diffusion. We can calculate the flux of attractant across the wave using Fick's first law:

$$\begin{aligned} J(s(t),t) &= -D_c \partial_x c(s(t),t) && (20) \\ &= -\frac{c_0}{\operatorname{erfc}\alpha}\sqrt{\frac{D_c}{\pi t}}\mathrm{e}^{-\alpha^2} && (21) \end{aligned}$$

The minus sign here only serves to tell us that the flux comes from a higher x position, and so we can ignore it. How many cells must the wave contain in order to degrade all the attractant it encounters? If each cell can degrade attractant at a maximum rate r, and the the number of cells required is N we can equate rN to the flux at the position of the wave,

$$rN = \frac{c_0}{\operatorname{erfc}\alpha}\sqrt{\frac{D_c}{\pi t}}\mathrm{e}^{-\alpha^2}. \qquad (22)$$

2.2 Decay of attractant profile in a bounded domain

Here we consider the profile of a chemoattractant, $c(x,t)$, stretching from a well containing cells at $x = 0$ to the end of the domain at $x = L$. We will assume that cells move very slowly relative to other processes, such that we need not update their position. The profile of the chemoattractant again changes according to Eqn (8), the diffusion equation.

We use variable separation in order to arrive at the Fourier series solution for this equation. We assume that the cell population is able to degrade all attractant that reaches it by diffusion very rapidly. This gives us the boundary condition $c(0,t) = 0$, restricting us to only the Fourier sine series. We also impose a zero-flux boundary condition $\frac{\partial c(L,t)}{\partial x} = 0$, restricting us to half-integer terms. Each remaining term in the series decays exponentially

in time. The solution is:

$$
\begin{aligned}
c(x,t) &= \sum_{n=0}^{\infty} A_n \sin\frac{(n+1/2)\pi x}{L}\exp\frac{(n+1/2)^2\pi^2 D_c t}{L^2}. \\
&= \sum_{n=0}^{\infty} A_n \sin\alpha_n x \;\; \mathrm{e}^{\alpha_n^2 D_c t}, \qquad (23)
\end{aligned}
$$

where $\alpha_n = (n+1/2)\pi/L$.

To determine the values of our A_n, we impose the initial condition of a square wave of amplitude c_0. This gives us a uniform profile, save for at $x = 0$.

$$
\begin{aligned}
c(x,0) &= \sum_{n=0}^{\infty} A_n \sin\alpha_n x \\
&= \frac{4c_0}{\pi}\sum_{n=0}^{\infty}\frac{1}{2n+1}\sin\alpha_n x, \qquad (24)
\end{aligned}
$$

$$(25)$$

therefore

$$A_n = \frac{4c_0}{(2n+1)\pi}, \qquad (26)$$

which we can substitute into Eq. 2 to give:

$$c(x,t) = \sum_{n=0}^{\infty}\frac{4c_0}{(2n+1)\pi}\sin\alpha_n x \; \mathrm{e}^{-\alpha_n^2 D_c t}. \qquad (27)$$

The total flux of chemoattractant onto the cells is again given by Fick's first law

$$
\begin{aligned}
J(x,t) &= -D_c\frac{\partial c(x,t)}{\partial x} \\
&= -\frac{2D_c c_0}{L}\sum_{n=0}^{\infty}\cos\alpha_n x \; \mathrm{e}^{-\alpha_n^2 D_c t} \qquad (28) \\
J(0,t) &= -\frac{2D_c c_0}{L}\sum_{n=0}^{\infty}\mathrm{e}^{-\alpha_n^2 D_c t}. \qquad (29)
\end{aligned}
$$

Or, equivalently, this can be written using the Jacobi theta function

$$J(0,t) = \frac{D_c c_0}{L}\theta_2(0, \mathrm{e}^{-\pi^2 D_c t/L^2}). \qquad (30)$$

As with the unbounded case, we can then relate the flux and the required population N with

$$rN = \frac{2D_c c_0}{L}\sum_{n=0}^{\infty}\mathrm{e}^{-\alpha_n^2 D_c t}, \qquad (31)$$

showing us again that the minimum number of cells needed in order to degrade all attractant decays over time.

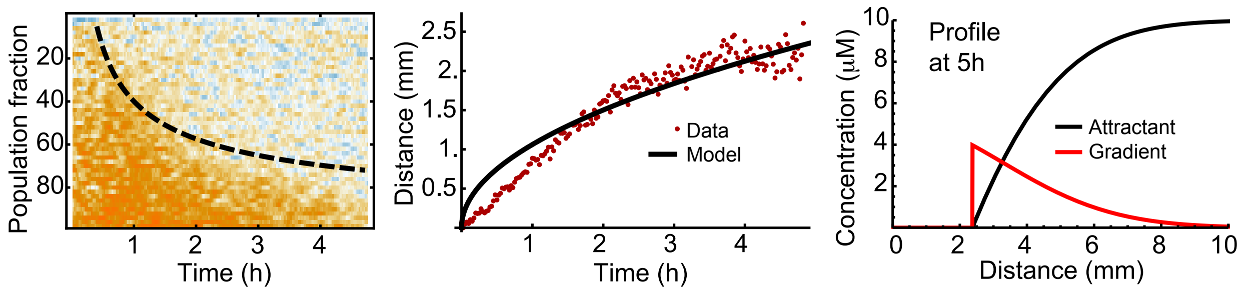

Figure S1: Model predictions and comparisons to data. (L) Predicted decay in the wave population overlayed on a heat map of the population's directed migration over time. (M) Real and model position of the wave. Here the real position for each frame is taken to be the position of cells moving away from the well, weighted by the speed of this directed movement. The model value for α s fitted to these data. (R) Attractant profile predicted by the simple model after 5h. The gradient shows us why we can expect to see a density increase in the wave, even without saturation- guidance cues are best at the back of the wave, where all attractant is degraded. Ahead of this point, guidance cues are poorer due to the lower gradient, and so the wave will catch up with any cells that get ahead.

2.3 Comparison of analytical and experimental results

In these analytical models, the parameter α controls the movement of the wave, such that when $\alpha = 0$, the wave is stationary at $x = 0$. When $\alpha \to 0$ and L is large there is (unsurprisingly) good agreement between the two models. Indeed, for $\alpha = 0$, $L = 30mm$ and $D_c = 12,000\mu m^2/s$, the required number of cells predicted by each model only differs by 0.25% over a simulation time of 5 days (though at this stage both models are, of course, unrealistic). As the contribution of α to the population of the wave happens entirely through the constant $\frac{1}{\operatorname{erfc}\alpha}\mathrm{e}^{-\alpha^2}$, over moderate time-scales the effect of migration is simply to increase the required population by a constant factor.

Fig. S1 shows the population, wave position and attractant profile predicted by the semi-unbounded model. Wave position is calculated by the average position of cells moving away from the well, weighted by their outward velocity. The model represents the population and position reasonably well, though the position of the wave does not match well at short times. We attribute this to limits on the speed and persistence of real cells not present in this idealised analytical model. The predicted gradient also demonstrates why the population density wave forms; cells ahead of the wave position experience a shallower gradient and by extension worse guidance cues. This means that they progess more slowly than the back of the wave does, and fall back into it.

3 The chemotactic fraction of a domain

A system has a point source of chemoattractant at $x = 0$ that maintains its concentration at c_0. It also has a sink at $x = L$, such that the steady state solution for the system is

$$c(x) = c_0(1 - x/L). \tag{32}$$

The general solution here is the sum of two solutions. One is the steady state solution, Eq. (11), the other is a Fourier series. For the latter, we apply the boundary conditions $c(0,t) = c(L,t) = 0$, restricting us to the integer harmonics of a Fourier sine series. We then find the corresponding Fourier components by equating it with the initial condition of the steady state solution minus a sawtooth wave of wavelength $2L$,

$$c(x,0) = c_0(1 - x/L) - \sum_{n=1}^{\infty} \frac{2c_0}{n\pi} \sin\frac{n\pi x}{L}. \tag{33}$$

The general solution we obtain is

$$c(x,t) = c_0\big((1 - x/L) - 2\sum_{n=1}^{\infty} \frac{1}{n\pi} \sin\frac{n\pi x}{L}\, \mathrm{e}^{-n^2\pi^2 D_c t/L^2}\big). \tag{34}$$

The expected receptor occupancy $\kappa(x,t)$ for a receptor at position x and t is

$$\begin{aligned} \kappa(x,t) &= c(x,t)/(c(x,t) + k_d) \\ &= \frac{c(x,t)/k_d}{(c(x,t)/k_d + 1)} \end{aligned} \tag{35}$$

and so the relative occupancy difference between the extreme ends of a cell of length l, centred at position $x > l/2$ is:

$$\Delta\kappa(x,t) = \frac{2\big(\kappa(x + l/2, t) - \kappa(x - l/2, t)\big)}{\kappa(x + l/2, t) + \kappa(x - l/2, t)}. \tag{36}$$

This is of course a generous simplification, as most receptors will not be at these extrema. We can use Eqs. 12 and 14 to calculate $\Delta\kappa(x,t)$ for any x and t. Zigmond gives the threshold value for chemotaxis as a relative difference in occupancy of 1%. We use this threshold to determine the fraction of the domain which can provide chemotactic guidance, the fraction of $x \in (0, L)$ for which $\Delta\kappa(x,t) \geq 0.01$. As $c(x,t)$ and k_d only appear in the ratio $c(x,t)/k_d$ it is clear that what determines guidance is the source concentration relative to receptor affinity, not either of these quntities alone. Similarly, as D_c and t only appear in the product $D_c t$, faster diffusion and longer waiting times are equivalent for transporting guidance cues from a point source. As such, we use these expressions as our axes for Fig. 8. Our numerical calculations assume that $l = 10\mu m$.

Symbol	Description	Value
D_f	The diffusion constant of folate	$12,000 \mu m^2$/min
D_{cAMP}	The diffusion constant of cAMP	$18,000 \mu m^2$/min
D_s	The diffusion constant of degrader	$4200 \mu m^2$/min
D_r	The diffusion constant of repellent	$1200 \mu m^2$/min
N	Line density of simulated cells	$1/\mu m$
r_{max}	The maximum rate of attractant degradation	1.85×10^{-6}nM/min
k_M	The Michaelis Menten constant of degradation	700nM
k_b	The chemotactic bias	0.5
l	The length of a cell	$10\mu m$
k_d	The receptor-attractant dissocication constant	20nM
σ	The circular standard deviation in direction	0.570rad/min
v_c	The speed of individual cells	20μm/min
k_{rec}	Rate of receptor replenishment (normalised)	0.002/min
r_{rec}	Rate of bound receptor degradation	0.02/min or 0.2/min

Table 1: Parameter table for chemotaxis simulations
